# Autophagy in Human T-Cell Leukemia Virus Type 1 (HTLV-1) Induced Leukemia

**DOI:** 10.3389/fonc.2021.641269

**Published:** 2021-03-31

**Authors:** Nicolás Ducasa, Daniel Grasso, Paula Benencio, Daniela L. Papademetrio, Mirna Biglione, Fatah Kashanchi, Carolina Berini, Maria Noé Garcia

**Affiliations:** ^1^ Instituto de Investigaciones Biomédicas en Retrovirus y SIDA (INBIRS), CONICET- Universidad de Buenos Aires, Buenos Aires, Argentina; ^2^ Cátedra de Fisiopatología, Facultad de Farmacia y Bioquímica, Universidad de Buenos Aires, Buenos Aires, Argentina; ^3^ Consejo Nacional de Investigaciones Científicas y Técnicas (CONICET), Instituto de Estudios de la Inmunidad Humoral (IDEHU), Buenos Aires, Argentina; ^4^ Cátedra de Inmunología, Facultad de Farmacia y Bioquímica, Universidad de Buenos Aires, Buenos Aires, Argentina; ^5^ Laboratory of Molecular Virology, School of Systems Biology, George Mason University, Manassas, VA, United States

**Keywords:** HTLV-1, autophagy, T-cell leukemia, NF-κB, tax

## Abstract

Viruses play an important role in the development of certain human cancers. They are estimated to contribute 16% to all human cancers. Human T-cell leukemia virus type 1 (HTLV-1) was the first human retrovirus to be discovered and is the etiological agent of adult T-cell leukemia/lymphoma (ATLL), an aggressive T-cell malignancy with poor prognosis. HTLV-1 viral proteins interact with mechanisms and proteins present in host cells for their own benefit, evading the immune system and promoting the establishment of disease. Several viruses manipulate the autophagy pathway to achieve their infective goals, and HTLV-1 is not the exception. HTLV-1 Tax viral protein engages NF-κB and autophagy pathways prone favoring viral replication and T cell transformation. In this review we focus on describing the relationship of HTLV-1 with the autophagy machinery and its implication in the development of ATLL.

## Introduction

Human T cell leukemia virus type 1 (HTLV-1), was the first human retrovirus discovered (1). It is the etiological agent of an aggressive T cell malignancy known as adult T cell leukemia/lymphoma (ATLL) and a neurologic disease named HTLV-1-associated myelopathy/tropical spastic paraparesis (HAM/TSP), other inflammatory syndromes, opportunistic infections, and lung diseases (2). HTLV-1 is transmitted through sexual contact, from mother to child (mainly by prolonged breastfeeding) and parenterally (3, 4). In 2014, HTLV-1 was included as group 1 human carcinogens by the International Agency for Research on Cancer (IARC) (5). The vast majority of HTLV-1 infected individuals are asymptomatic and around 3-5% of them will develop ATLL, that usually occurs after a long latency period. It is clinically classified as smoldering, chronic, lymphoma and acute (6). The smoldering and chronic without unfavorable prognostic factors are categorized as indolent ATLL and generally progress slowly. On the contrary, acute, lymphoma and chronic with unfavorable prognostic factors are aggressive forms and patients have a survival of months (7, 8). The HTLV-1 genome shares the structural features of other retroviruses, but it also has a pX region which encodes regulatory proteins, such as Tax and bZIP factor (HBZ) (9). HBZ and Tax have opposing functions in most transcription pathways, but both proteins play a critical role in HTLV-1 infection as well as in growth and survival of leukemia cells (10).

Autophagy (also known as macroautophagy) is a degradative process for cellular components including macromolecules such as proteins, RNA and even whole organelles (11). Under stress conditions such as cell starvation, inhibition of mTORC1 (mammalian Target of Rapamycin Complex 1) leads the activation of ULK1 (unc-51 Like Autophagy Activating Kinase 1) complex which in turn triggers the autophagosome biogenesis (12). ULK1 activates a complex which includes BECN1 among its members and a PI3KC3 (phosphatidylinositol 3-kinase Class 3). The PI3P (phosphatidylinositol 3-phosphate) generated by this last complex is required for recruitment of further autophagic proteins and the autophagosome formation. Autophagosomes are double membrane vesicles decorated by LC3 protein and, once it is loaded with the cargo, this particular vesicle is carried to fuse with lysosomes where the cargo is eventually degraded (12–15).

Alternatively, autophagosomes can fuse with components of the endosomal system, late endosomes or multivesicular bodies (MVBs) (16). These hybrid compartments, named as amphisomes, have the options of degrading its intravesicular material by fusion with a lysosome or fusing with the plasma membrane (17, 18). In the case they fuse with the plasma membrane, their contents are released outside the cell including extracellular vesicles (EVs) (19, 20). These small vesicles have recently gained special relevance since they are important intercellular messengers capable of carrying several molecules, proteins, nucleic acid, and even viral components, and yield an effector response in the target cell (21–24). Importantly, exciting new data is supporting the idea of a superlative crosstalk between autophagy and EVs machineries (25–30). Moreover, autophagy can take an antiviral or pro-viral role. It could be expected to degrade intracellular pathogens, but certain viruses have evolved to use the autophagic machinery for their own benefit, increasing viral replication and viral spread (31).

## Adult T-Cell Leukemia/Lymphoma

The adult T-cell leukemia/lymphoma or ATLL is a malignant and aggressive neoplasm as a consequence of HTLV-1 infection. In endemic regions of Japan, ATLL affects about 8.7 persons per 10,000 HTLV-1 infected citizens and, having in mind an annual incidence of 20 million around the world, it is expected a significant number of persons suffering this pathology (32). In Latin America, 1% of HTLV-1 infected individuals are asymptomatic positive, but in some endemic areas it might reach 10% (32). It is interesting to know that HTLV-1 infects T cells, B cells, fibroblasts, dendritic cells, and macrophages, though hitherto data show it is only capable of transforming regulatory T cells which are positive for CD4/CD25 (33). The carcinogenesis induced by HTLV-1 infection possesses a biphasic behavior, with an initiation and a maintenance phases. Epidemiology demonstrates that ATLL onset is observed about the fifth decade in individuals that were infected during the firsts years of life (34, 35). This suggests an extensive latency period coincident with the conception that oncogenesis is initiated in a first phase of viral infection and then a second phase where oncogenic properties of transformed cells are maintained (34, 35).

HTLV-1 seems to rely on two main proteins for cellular transformation, HBZ and Tax. Data suggests that HBZ is important for viral replication, cellular proliferation and evasion from the immune system (36, 37). Then, the major key role of HBZ in oncogenesis is maintaining the oncogenic phenotype by attenuation of host immune response against leukemic cells and fostering a microenvironment appropriate for HTLV-1 infected cells (38). On the other hand, Tax is the main actor for the T cells transformation process engaging several cellular pathways (39). This means that Tax appears early during infection and during the long period of latency time, and it is crucial to initiate cellular transformation. Once cells are transformed, HBZ enters in the second phase for maintaining the transformed phenotype. This is also supported by the fact that Tax gradually disappears during that time, to the point of being almost undetectable, contrary to HBZ whose presence is prominent and ubiquitous in advanced stages of ATLL (40, 41).

The central role of Tax in ATLL is highlighted with the observation that impairment of functionality of *tax* gene impedes T cells transformation (42). Even more, Tax overexpression provokes a leukemia phenotype in transgenic mouse models (43, 44). This data suggests that Tax is indeed enough for T cells immortalization. Moreover, the capacity of Tax to induce cell survival, proliferation and bypass tumor suppressor processes such as senescence and apoptosis has been vastly demonstrated (45–50). The powerful property of Tax lies on its ability to activate a myriad of signaling pathways including PI3K/AKT, p53 inhibition, induction of ROS (reactive oxygen species) production and even genome instability by direct DNA damage and impairment of proteins related to DNA repair (51, 52). Although, those characteristics are important in Tax-mediated transformation is worthy to mention specially the NF-κB pathway. Tax has the ability to activate both canonical and noncanonical NF-κB pathways which in turn set up a broad cell survival program (53–55). Tax-mediated initial activation of NF-κB pathways is in such a way that it persists even after Tax expression has disappeared (52, 56).

## Htlv-1 Tax Relationship With The Nf-κb Pathway

The NF-κB family of transcription factors is composed of five members: RelA (p65), c-Rel, RelB, NF-κB1 (p50) and NF-κB2 (p52), which can form hetero or homodimeric combinations (57). There are two major pathways for NF-κB activation: the canonical and non-canonical NF-κB signaling pathways. Canonical NF-κB signaling is induced upon stimulation by pro-inflammatory cytokines, such as TNF-α, IL-1β and IL-6, pathogen-associated molecular patterns (PAMPs) from viruses and bacteria, agonists for the B or T cell antigen receptors (BCR or TCR), and chemicals or radiation (58). On the other hand, non-canonical NF-κB signaling pathway is restricted to a subset of TNF family members such as B cell activating factor (BAFF), lymphotoxinβ-(LTβ) and CD40L (59). These two pathways of NF-κB activation differ, not only in the involved receptors, but also, in the implicated molecules and the generated response. The induction of the canonical NF-κB signaling involves a variety of different adaptor molecules to engage the IKK complex which in turn triggers the signaling pathway (60). IKK complex consists of the regulatory subunit IKKγ/NEMO, and IKKα and IKKβ, the catalytic ones (61). Once activated, IKK phosphorylates IκBs subunits (IκBα, IκBβ and IκBϵ) inducing the IκBs ubiquitination and proteasomal degradation (62). Then, classical NF-κB dimers, like p50/RelA and p50/c-Rel, are released from IκB to enter the nucleus and induce the transcription of target genes (60). This activation of the canonical NF-κB pathway under physiological conditions, induces a rapid but transient transcriptional response (58, 59). On the contrary, non-canonical NF-κB signaling activation relies on NIK, which in resting cells is constantly degraded by an E3 ligase complex consisting of the E3 ligases c-IAP1/2 and the adaptor TRAF3/TRAF2. Activation of BAFFR, LTβR and CD40 provokes inactivation of the TRAF/c-IAP complex and the consequent NIK stabilization. In this situation, NIK phosphorylates IKK, which in turn phosphorylates p100/RelB tagging it for proteasomal processing and the consequent release of p52/RelB, which translocates to the nucleus. Compared to the canonical way, non-canonical NF-κB response is delayed, but its transcriptional response is sustained in time (58, 59). It has been described the existence of negative regulators of the NF-κB pathway that could be involved in the constitutive activation of NF-κB in ATLL such as TNF-α-induced protein 3 (TNFAIP3, A20), Cylindromatosis (CYLD), and NSFL1 cofactor (p47) among others. The implication of any of these negative regulators of the NF-κB pathway could be of extreme importance in the persistence of its activation (63–65).

**Figure 1:**
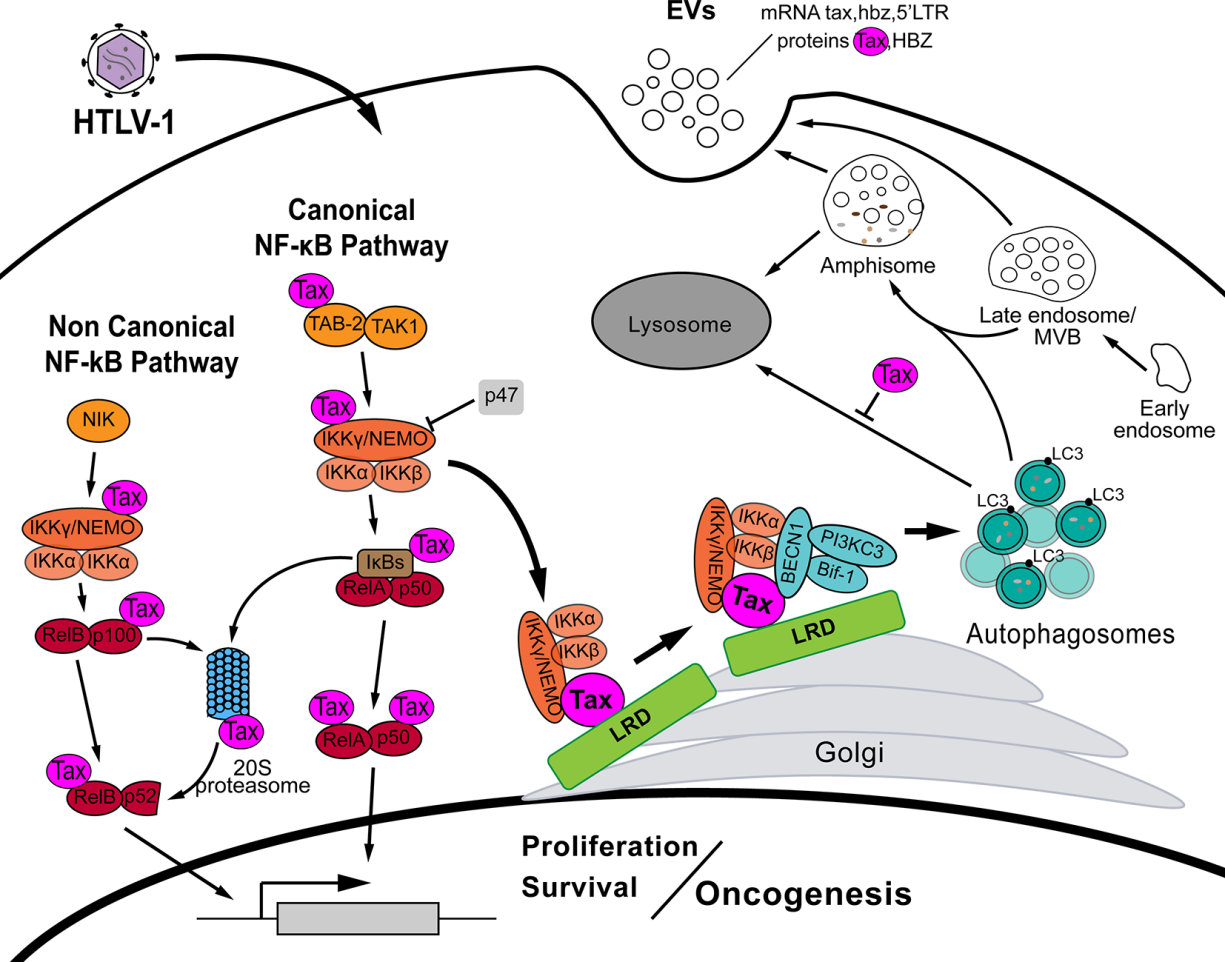
In HTLV-1 infection, the viral protein Tax interferes at several steps of both canonical and noncanonical NF-κB pathway in order to activate it, inducing cell survival and proliferation, and eventually resulting in oncogenesis. By interaction with IKKγ/NEMO, Tax recruits and activates IKK complex (IKKγ/NEMO, IKKα, IKKβ) in lipid raft domains (LRD) on the Golgi. After IKK activation, Tax recruits the autophagy proteins BECN1, Bif-1 and the PI3KC3 complex through its direct interaction with BECN1, which in turn binds also with IKKα, IKKβ. Then, Tax deregulates the autophagy pathway fostering autophagosomes biogenesis but, at the same time, blocking the autophagosome-lysosome fusion. Autophagosomes accumulation enhances HTLV-1 replication. Moreover, recent data suggest a crosstalk between autophagic and extracellular vesicles (EVs) biogenesis pathways. EVs from HTLV-1 infected cells bearing the viral proteins Tax and HBZ among some host proteins, and transcriptional mRNA of Tax, HBZ and 5’LTR has been reported.

In HTLV-1 infection, Tax persistently activates both canonical and non-canonical NF-κB pathways which are required for cell survival and T lymphocyte transformation (66, 67). Indeed, a persistent NF-κB activity is observed in HTLV-1 transformed cell lines (54). By intervention at different levels, Tax ensures the NF-κB pathway activation without external signals. HTLV-1 Tax interacts with TAK1-binding protein 2 (TAB-2) activating MEKK1 and TAK1 which in turn activate the IKK complex (68, 69) (**Figure 1**). The direct association of Tax with IKKγ/NEMO in the lipid raft domains (LRD) localized on Golgi is key for Tax goals (70–73). This Tax-IKKγ/NEMO interaction recruits the whole IKK complex and this action is indispensable for its activation (**Figure 1**) (74–76). Moreover, the resulting degradation of IκBs and consequent release of NF-κB transcription factor subunits are further enhanced by Tax direct interaction with IκBs and 20S proteasome (54, 77) (**Figure 1**). On the other hand, Tax also interacts with IKKγ/NEMO and p100 to induce the proteasome-mediated processing of this last in order to activate the non-canonical NF-κB pathway (55, 78–82). These data are supported by the fact that IKK is persistently activated in primary ATLL and HTLV-1 transformed cells (54, 77). The deep involvement of Tax with the NF-κB pathway is justified by the fact that the activity of this pathway is indispensable for T cell transformation and the maintenance of the leukemic phenotype (81, 83).

## Htlv-1 Tax Deregulation Of Autophagy

The fight between cells and viruses came from a constant competitive evolution. Cells have developed several strategies against viral infections and autophagy is into their toolkit repertory. For instance, xenophagy and virophagy are two types of selective autophagy that are activated in order to clear intracellular pathogens (84, 85). Xenophagy leads cells to microorganisms recognition, including viruses, to target them towards lysosome, through the autophagy machinery for degradation by the lysosomal hydrolases (84). In a similar way, virophagy tags specific viral components to be degraded by the autophagy flux (86). Nevertheless, viruses also have evolved to evade those strategies and indeed use autophagy machinery for their own benefit (87). In dendritic cells, one strategy of human immunodeficiency virus (HIV-1) is enhancing mTORC1 activation which in turn inhibits the autophagy pathway (88). Another way, used by herpes simplex virus type 1 (HSV-1), is to produce a specific viral protein that suppresses autophagy by its binding to BECN1, which is essential for autophagosome biogenesis initiation (89). Furthermore, most RNA viruses such as hepatitis C virus (HCV) induce autophagy flux to use the double membrane of autophagosome vesicles to hide themselves and, in fact proliferate and come out from the host cell (87, 90, 91). All in all, viruses have developed several strategies with the goal of modifying autophagy in each step, avoiding cellular defensive mechanisms and promoting their proliferation.

It is clear that HTLV-1 infection induces cytoplasmic autophagosomes accumulation and indeed this event increases viral particles production, measured by the viral capsid protein p19 (92). The single transfection of Tax in HeLa and Jurkat cells is enough to accumulate cytoplasmic LC3 positive dots (58). Interestingly, Tax co-localizes with cytoplasmic LC3 puncta but its capacity to accumulate autophagosomes is highly increased when cells are transfected with a Tax targeted by myristoylation to LRDs (83). Worth to note, HTLV-1 capacity to increase cytoplasmic autophagosomes relies also on Tax ability to activate the NF-κB pathway (92). For instance, a mutated Tax without the ability to activate IKK complex is also unable to induce autophagy (83, 93). Besides, impairment of any member of the IKK complex, by abolition of the catalytic activity of IKKα or IKKβ, or the knockdown of IKKγ/NEMO, decreases the cytoplasmic LC3 positive autophagosomes (83). As commented above, Tax activates the IKK complex by recruiting IKKγ/NEMO, and the IKK complex, to the LRD located at the Golgi (71, 73). Additionally, in those LRD, Tax recruits BECN1 and Bif-1, and indeed there is an interaction with PI3KC3 (56, 83). All those proteins belong to the autophagy PI3KC3 complex, the first structural complex of the autophagosome biogenesis (94). In Tax-immortalized T cells Tax co-precipitates with BECN1 and PI3KC3 but not with UVRAG which form with BECN1 another complex related to the autophagosome maturation (95, 96). Without BECN1, Tax is unable to co-precipitated PI3KC3 suggesting that the interaction is through BECN1. Moreover, the sequence of BECN1 that goes from aminoacids 250 to 300 is implicated in the BECN1-Tax interaction (96). Worthy, Tax-mediated recruitment and subsequent activation of IKK complex in the LRDs is a prerequisite to further recruitment of BECN1 and Bif-1 forcing the activation of those autophagy proteins to trigger the autophagosomes biogenesis. This process seems to be exclusive of HTLV-1 infected cells because the co-distribution in LRDs of IKK complex with BECN1 and Bif-1 is only observed in Tax expressing cells (83). Furthermore, the entire IKK complex is key in this Tax-mediated autophagy dysregulation since Tax does not colocalizes in the LRD in absent of IKKγ/NEMO and depletion or impairment of any of the three IKK complex members impedes BECN1 and Bif-1 recruitment to the LRD. The importance of this recruitment is highlighted by the fact that myristoylation of either BECN1 or Bif-1 to target them towards the LRDs induces autophagy (83). It is important to consider the key role that this function of Tax over autophagy seems to have for HTLV-1 infection. Wang and colleagues described how Tax-mediated autophagy provides to infected cells resistance to cell death and, in fact, they suggest to explore autophagy inhibition as a possible treatment against HTLV-1 infection (93).

The relationship between HTLV-1 infection and the autophagy pathway is intricate and the roles of Tax/IKK over the autophagy proteins go in both directions. BECN1 is needed to maintain NF-kB and STAT3 activity in HTLV-1 infected cells (96). STAT3 cooperates with NF-κB in HTLV-1 infected cells. When silencing BECN1, in HTLV-1 transformed cells, a decreased NF-κB and STAT3 activity as well as an impairment in cellular growth is observed (96). Furthermore, PI3KC3 or BECN1 depletion significantly slows the proliferation of HTLV-1 infected T lymphocytes (83). Co-precipitation experiments show that BECN1 interacts directly with the catalytic subunits of IKK complex (i.e. IKKα and IKKβ) through its C-terminal 150 amino acids region. Neither IKKα nor IKKβ alone can co-precipitate BECN1 and the PI3KC3 complex suggesting that both are indispensable for the interaction (60). Altogether, in the LRDs Tax recruits the IKK complex, by its interaction with IKKγ/NEMO, and the autophagy PI3KC3 complex by its interaction with BECN1. In that context BECN1 interacts with IKKα and IKKβ and it might suggest that LRDs function as a platform where Tax engages the NF-kB and autophagy pathways by the local interaction of Tax, BECN1 and IKK complex (**Figure 1**). Furthermore, as the autophagy is required for the maintenance of NF-κB activity and the LRD recruitment and activation of IKK is needed for Tax-mediated autophagosomes biogenesis, it is logical to speculate about a positive feedback loop between NF-κB and autophagy pathways in HTLV-1 infection (**Figure 1**).

Beyond all above comments, HTLV-1 possesses other goals for autophagy deregulation. The p47 protein was recently found in an attempt to find IKKγ/NEMO interactors. Interestingly, p47 is found highly decreased in HTLV-1 infected cells and in cells from ATLL patients (97). Among its different functions, p47 with its UBA domain is related to degradation of ubiquitinated proteins (98, 99). In CD4+ T lymphocytes, p47 recognizes ubiquitinated IKKγ/NEMO and induces its lysosomal degradation since lysosomal inhibitor but not MG132 (proteasome inhibitor) restore the IKKγ/NEMO levels (97). In this way, p47 negatively regulates IKKγ/NEMO independently of the other two known regulators of the IKK complex, A20 and CYLD (97, 100). Worth to note, shRNA-mediated depletion of p47 significantly potentiates IκBα phosphorylation induced by transfection of Tax in HeLa cells (97). This means that p47 opposes the action of Tax over the NF-κB pathway. In fact, in co-precipitation assay p47 is unable to precipitate IKKγ/NEMO in presence of Tax suggesting that Tax disrupts the p47 binding to IKKγ/NEMO. The regulation of p47 seems to be mediated by its stability since cells from patients with the acute type of ATLL the expression of p47 is similar to uninfected CD4+ T lymphocytes but the amount of p47 protein is significantly lower (65). This degradation of p47 is avoided upon lysosomal inhibition but not with MG132. Moreover, in MEFs Atg5^+/+^ the induction of autophagy by starvation reduces the levels of p47 in stark contrast to Atg5^-/-^ MEFs where the lack of autophagy does not perturb p47 levels upon cell starvation. Finally, similar results were obtained in HTLV-1 infected cells where shRNA-mediated depletion of ATG5 increased the amount of p47, and concomitantly a decrease in IKKγ/NEMO, phosphorylated IκBα and even CADM1 (which is a receptor dependent on NF-κB activity) was detected (65). All these data confirm the degradation of p47 by the autophagy pathway and give an additional reason for Tax-mediated deregulation of the autophagy pathway (**Figure 1**).

The Tax-mediated deregulation of autophagy by Tax/BECN1/IKK in the LRDs is completed with its effects on late steps of autophagosome maturation. Data shows that inhibition of autophagosome-lysosome fusion, by means of bafilomycin A1, improves Tax stability suggesting that Tax could be degraded into the lysosome through the autophagy pathway (92). In consequence, they proved that Tax inhibits the fusion of those degradative vesicles (92). Then, HTLV-1 Tax exerts a deep interference in the autophagy pathway fostering autophagosomes biogenesis but, at the same time, inhibiting the autophagosome-lysosome fusion (**Figure 1**). Additionally, new points of contacts between autophagy and HTLV-1 Tax are still being described such as the case of SQSTM-1/p62. In MEFs and HEK293T cells, but not in Jurkat cells, depletion of SQSTM-1/p62 impair the Tax-mediated NF-κB activity. Indeed, SQSTM-1/p62 directly interacts with Tax in the Tax/IKK complex located in Golgi-associated structures (101). SQSTM-1/p62 is an autophagy receptor with domains for recognition of ubiquitin chains and LC3 to canalize cargoes towards the autophagy-mediated degradation (102, 103). Similar data is obtained with Optineurin, another autophagy selective receptor, but interestingly in both cases Tax interaction with those proteins seems to be related to Tax-mediated NF-κB activation and not with Tax degradation (101, 104). By the side of HBZ, it negatively regulates the autophagy pathway. In cytoplasm, HBZ associates and inhibits GADD34 which has been demonstrated to be a mTOR inhibitor. Then, HBZ enhances mTOR activity probably for allowing its anabolic functions, though mTOR inhibits the autophagy triggering and in consequence HBZ indirectly inhibits the autophagy pathway (105, 106). This is interesting because it might be related to the strategy used by Tax to induce autophagy that is going directly to BECN1/PI3KC3 complex in a manner independent of mTOR activity status. All in all, these data demonstrate that we are not yet watching the whole panorama. Future work would shed light about the complex mechanisms in Tax-autophagy close relationship and whether it includes other autophagy related processes such as selective autophagy, non-canonical forms of autophagy, etc.

Going even further, it has been demonstrated that a constitutively activated IKK complex induces autophagy *in vitro* and *in vivo* (107). IKK is implicated in early carcinogenesis inducing autophagy in several tumors in order to cope with the stress related to tumor microenvironment (108). IKKβ seems to be crucial in this intricate mechanism since this molecule transactivates BECN1 to induce autophagy (109). With very interesting data, Peng and colleagues show that IKKβ induces accumulation of autophagosomes, but at the same time enhances the fusion of those autophagic vesicles with the MVBs, resulting in amphisomes (110). They also observed the IKKβ-mediated driving of amphisomes toward the plasma membrane with the consequent release of small extracellular vesicles (EVs) which are positive for the autophagic proteins LC3 and SQSTM-1/p62 (110). Importantly, Tax has recently been found in EVs from HTLV-1 infected T cell lines (111). Moreover, those EVs bear the viral proteins Tax and HBZ among some host proteins, and transcriptional mRNA of Tax, HBZ and 5’LTR (**Figure 1**) (111). The incubation of those EVs with uninfected cell cultures (CTLL-2 and PBMC) increases survival under stress conditions (111, 112). This was further confirmed in EVs from ATLL patients derived leukemia cells where Tax was also detected (113). In the same work, EVs purified from ATLL cell line HUT-102 were taken up by bone marrow mesenchymal stem cells (MSC) with the consequent activation of NF-κB pathway, observable morphological changes, proliferation, activation of a migratory phenotype and presence of angiogenic markers (**Figure 1**) (113). Putting together the effect of IKKβ over the autophagy pathway and the release of EVs with presence of EVs containing Tax from infected cells and ATLL patients cells it is not difficult to speculate that both events might be connected, though it needs to be confirmed. For sure, these results broaden the views about the possible roles of Tax, and/or other HTLV-1 proteins, regarding all these pathways. Finally, we are just observing the tip of the iceberg about HTLV-1, autophagy, and their relationship in the development of ATLL.

## Conclusions And Perspectives

Most viruses have developed different strategies to overcome cell defenses over evolution, and even more, to use those cellular mechanisms for their own viral cycle. Autophagy is an important homeostatic cellular process and as such it has an antiviral program of action like virophagy and xenophagy. Indeed, HTLV-1 virus induces autophagy to foster viral production. Tax protein seems to be the wild card weapon of HTLV-1, which is able to orchestrate most of the viral action to success in its infective attempt. In the same movement, Tax engages the autophagy and the NF-κB pathways in such a way that it is enough to produce the oncogenic transformation of the cell and, indeed, go on even when Tax is not more detectable. The recent results around IKK, autophagy, the vesicular trafficking and the EVs carrying Tax let us imagine that this is just the beginning in our comprehension of this intricate process. Finally, during HTLV-1infection, Tax is in the middle of a complex crossroad that includes inflammatory signal pathways, apoptosis, autophagy, and intercellular communication, that could be the key to uncover its oncogenic transformation ability.

## Author Contributions

CB, DG, and MG conceived the ideas. ND, DG, CB, and MG collected literature resources. DG and MG wrote the original draft. PB, MB, and FK reviewed manuscript. ND, DP, CB, DG, and MG reviewed and edited the manuscript. All authors contributed to the article and approved the submitted version.

## Funding

This work was partially supported by UBACYT-UBA-(20020190200047BA) (DLP), UBACYT-UBA-(20020190200293BA) (CB), PICT 2015-1105 - ANPCyT (MNG), PICT 2018-02220 - ANPCyT (DG), PICT 2019-00736 - ANPCyT (DP) and PICT 2019-00433 - ANPCyT (CB). The authors thank the University of Buenos Aires and the National Council for Scientific and Technological Research (CONICET) for the facilities to carry out our work every day.

## Conflict of Interest

The authors declare that the research was conducted in the absence of any commercial or financial relationships that could be construed as a potential conflict of interest.
